# Costs of breast cancer care by stage and HER-2 Status: a population-based study

**DOI:** 10.3389/frhs.2026.1811420

**Published:** 2026-06-03

**Authors:** Alessandra Buja, Massimo Rugge, Marcello Di Pumpo, Fabio Girardi, Claudio Palmeri, Stefano Guzzinati, Rocco Cappellesso, Eliana Ferroni, Emanuela Bovo, Manuel Zorzi, Valentina Guarneri

**Affiliations:** 1 Department of Cardiological, Thoracic, Vascular Sciences and Public Health, University of Padua, Padua, Italy; 2 Department of Medicine – DIMED, Anatomic Pathology and Cytopathology Unit, University of Padua, Padua, Italy; 3 Department of Life Sciences and Public Health, Università Cattolica del Sacro Cuore, Rome, Italy; 4 Department of Prevention, AULSS6 Euganea, Padua, Italy; 5 Veneto Institute of Oncology IOV—IRCSS, Padua, Italy; 6 Veneto Tumor Registry - RTV, Azienda Zero, Padua, Italy; 7 Institute of Anatomic Pathology, Padua University Hospital, Padua, Italy; 8 Dipartimento di Scienze Chirurgiche, Oncologiche e Gastroenterologiche, University of Padua, Italy

**Keywords:** breast cancer, cancer costs, cancer registry, cost analysis, economic analysis, Epidemiology

## Abstract

**Introduction:**

Breast cancer (BC) is one of the most common and leading causes of cancer-related deaths among women. Cost analysis of BC treatment provides essential information for health policy planning, including resource allocation. This study aims to present real-world data on the cost components of BC care, categorized by cancer stage and HER2 status.

**Methods:**

This retrospective, population-based cohort study examined 1,232 new BC cases diagnosed in 2021 across three Local Health Units in the Italian northeastern Veneto Region. The study calculated per-patient costs for all three years following BC incidence. Care costs were categorized by items and analyzed by cancer stage and HER2 BC status.

**Results:**

Over a three-year post-diagnosis period, care costs increased from € 25,630 to € 90,924. Total hospitalization costs increased from €6,500 in stage I to €15,488 in stage IV, with a ratio of 2.4. Outpatient costs increased from €11,143 to €25,296, with a ratio of 2.3. In-hospital drug administration rose from €4,427 to €34,210, with a ratio of 7.7. Across all stages, the mean overall cost increased from €27,817€ in HER2-negative patients to €52,193€ in HER2-positive patients, while the breast cancer–specific cost increased from €17,881€ to €36,564€, respectively. HER2-positive cancers incurred almost twice the cost of HER2-negative BC.

**Conclusions:**

The overall cost of BC care increases with the stage of cancer at diagnosis, which also affects the allocation of costs among various cost components. In the early stages, expenses mainly relate to surgical and outpatient procedures. For more advanced BC, care costs rise substantially, mainly due to systemic therapies and hospital-based care. The HER status at cancer presentation significantly impacts care costs across all cancer stages.

## Introduction

Breast cancer (BC) is the second most prevalent malignancy, responsible for approximately 2.3 million new cases. It ranks as the fourth leading cause of cancer-related mortality, with an estimated 666,000 deaths worldwide in 2022 (1). The stage at the time of diagnosis significantly influences clinical outcomes and economic implications: early-stage breast cancer (stages I–II) is associated with five-year survival rates exceeding 90%, whereas advanced stages (stages III–IV) are linked to substantially poorer prognoses, with survival rates declining to approximately 30% for metastatic disease (2). This epidemiological significance underscores substantial health concerns and exerts considerable pressure on public healthcare systems. A comprehensive evaluation of BC-associated care costs is imperative for informing health policy development and guiding the strategic allocation of financial resources within both public and private healthcare sectors systems (3–6).

The stage of cancer at the time of initial diagnosis is a crucial determinant in accurately estimating the cost of breast cancer treatments (7). Well-established evidence shows that treatment costs consistently rise with the cancer stage at diagnosis (5, 8). A comprehensive review found that the average economic burden of treatment increased by 32%, 95% and 109% for stages II, III, and IV, respectively, compared to stage I (9). In 2024, Mittmann et al. found an average incremental annual cost of 20,194 US$ (27,485 CAD) per patient, ranging from 11.453 US$ (15,588 CAD) for stage I to 100,895 US$ (137,319 CAD) for stage IV (8). An English study focusing on the first year of treatment reported an average hospital-related cost of 7,033 US$ (£ 5,167) for stage I, 10,363 US$ (£ 7,613) for stage II, and 18,145 US$ (£ 13,330) for stage IIIA (10). However, the costs associated with the management of breast cancer patients vary moderately across different countries. A study conducted in a third-level medical facility at the Mexican Social Security Institute found that the average cost of breast cancer care per patient is 5,231 US$ and 7,790 US$ for early and advanced stages, respectively (*p* = 0.024) (11). Similarly, the average direct medical cost of breast cancer management in Benin was estimated at 476 US$ (±775 US$) (12). A systematic review found that annual direct medical costs ranged from 3,807 to 10,052 US$. Drug cost is the most important component of direct medical costs, accounting for 42.4% to 88.2% of total costs (13). Precision medicine has transformed treatments for breast cancer and their associated costs. In 2020, Brandão and colleagues emphasized the influence of biological cancer profiles on the expenses of innovative therapies. Concentrating on stages I, II, and III, this Portuguese study demonstrated that the median cost of care was fourfold higher in patients with HER2 + tumors compared to those with HR+/HER2− and triple-negative cancers (14–16).

Most contemporary data primarily associate treatment expenses with the stage of neoplastic disease and less frequently with molecular specimens. Nevertheless, a dependable cost assessment should encompass all care-related components. Furthermore, it is crucial to identify all factors that substantially influence the overall cost of cancer treatment, beyond hospitalization and pharmaceutical costs. Aspects such as outpatient consultations, laboratory examinations, imaging procedures, medical devices, and palliative care offer a more comprehensive understanding of the healthcare burden, thereby providing a more precise representation of the total financial impact.

This study aims to estimate the average direct medical costs of breast cancer treatments, categorized by cancer stage and HER-2 status. Using regional population-based cancer registry records, all BC patients were systematically linked to various regional administrative databases recording a variety of healthcare procedures and services use. This linkage generated high-quality real-world data on the costs associated with itemized multimodal management strategies for breast malignancies.

## Methods

### Healthcare setting

The Italian National Health System is funded by general taxes and administered regionally, in accordance with national guidelines. The public health system is founded on ethical principles such as universal coverage, fairness, unrestricted access, freedom of choice, and diversity of services. Regional authorities oversee healthcare management. Veneto is a northeastern Italian region with a population of 4.85 million. The Veneto population-based cancer registry [*Registro Tumori Veneto* (RTV)] monitors cancer cases across the entire regional population.

In 2013, the Regional Government established a network of health institutions committed to preventing and treating cancer diseases [*Rete Oncologica Veneta* (ROV) (12, 17)]. A multidisciplinary committee, comprising specialists in the most common oncology diseases, was tasked with developing, implementing, monitoring, and updating diagnostic and therapeutic pathways for all significant oncology diseases. The guidelines provide strategies for cancer prevention, diagnostic procedures, treatment options, end-of-life care, and palliative care.

In 2022, the Regional Oncology Network released the Breast Cancer Diagnostic and Therapeutic Care Pathways (PDTA). The original document and its updates detail BC clinical management, including prevention strategies, diagnostic procedures, treatments, and end-of-life support (13, 18).

### Study population

This study adopts a retrospective, population-based cohort design involving 1,232 participants. All new BC cases diagnosed in 2021 across three Local Health Units (LHUs: ULSS 2, ULSS 6, and ULSS 8) of the Veneto region (14, 15, 19, 20), no exclusion criteria were applied. Cancer recording covered all residents of the three considered LHUs. The clinical-pathological BC data were obtained from the population-based Regional Cancer Registry (RTV). Cases with inconsistent TNM staging information were categorized as indeterminate for stage and labeled “missing stage” for analytical purposes.

HER2 status was initially evaluated using immunohistochemistry. Cases with HER2 = 0 or HER2 1 + were excluded from any molecular targeted therapy. To assess BC patients’ eligibility for targeted therapy, HER2 2 + cases underwent fluorescence *in situ* hybridization (FISH) testing for HER2 amplification. Only cancers that tested IHC 3 + or FISH-positive were considered HER2-positive and received targeted treatment.

### Overall and cancer-specific costs analysis

Economic analyses were performed using anonymized administrative data obtained from the Regional Health Authority. All patients were identified by an anonymous code linking them to their administrative records. The three-year per-patient cost analysis distinguished between two categories: a) overall expenditures related to patients’ access to healthcare services, regardless of the procedure type or clinical reason; and b) cancer-specific clinical interventions directly related to cancer clinical pathways defined by the Regional Oncology Network (ROV) (12, 17) during the three years following the initial diagnosis.

To prevent bias from patients who died during the observation period, the average costs were adjusted based on the number of days each patient survived within the three-year follow-up.

Table 1 describes the profiles of the collected administrative data.

**Table 1:** Regional administrative databases used in the cost estimates for the diagnosis and treatment of breast cancer patients. In accordance with the aims of this study, costs related to outpatient drug distribution were separated from those associated with in-hospital drug administration.

ADMINISTRATIVE DATABASES	DATA COLLECTION
HOSPITALIZATION	Refers to global costs for each hospital admission based on Diagnosis-Related Groups (DRGs) categorized according to the Italian inpatients’ formulary (*i.e.*, *Tariffario Prestazioni Ospedaliere*).
OUTPATIENTS’ PROCEDURES/SERVICES	Refers to global costs of the procedures/services provided by the Regional Health Service at outpatient facilities.
EMERGENCY ROOM ADMISSIONS	Refer to global costs for all medical procedures and interventions performed at the Accident and Emergency department.
OUTPATIENT DRUGS DISTRIBUTION IN HOSPITAL DRUG ADMINISTRATION	Refers to global costs of medical therapies calculated by multiplying the drug unit cost by the prescribed quantity.
MEDICAL DEVICES	Refers to the global costs related to prosthetic and other clinical devices.
HOSPICE ADMISSION	Refers to global hospice-related costs calculated by multiplying the daily rate by the length of stay.

At the patient level, expenses were further categorized by disease stage at diagnosis and HER2 status.

Record linkage procedures, cost calculations, and descriptive statistics computations were all performed using the statistical software R (version 4.3.2).

### Ethics

This study was conducted in accordance with the principles outlined in the Declaration of Helsinki. All data were anonymized in compliance with Italian regulations and managed for quality assurance and monitoring. The data analysis was performed on aggregated anonymous data, ensuring that individuals could not be identified.

## Results

The study included 1,232 female patients (mean age 65.08 ± 14.31 years) who received an initial BC diagnosis in 2021. The clinicopathological characteristics of the study population are summarized in Table 2.

**Table 2:** Clinicopathological characteristics of 1,232 breast cancer patients.

Tumor status (T)		Age		pTNM stage		Grading	
T0	0.6% (7)	Mean (sd)	65.08 (14.31)	Stage I	43.8% (540)	Differentiated or well differentiated	12.9% (159)
T1	59.9% (738)	Median (IQR)	65.15 (53.18 - 76.49)	Stage II	23.5% (290)	Moderately differentiated	52.8% (651)
T2	28.2% (347)	<45	7.1% (87)	Stage III	4.5% (56)	Poorly differentiated	30.1% (371)
T3	4.2% (52)	45-59	31% (382)	Stage IV	5.1% (63)	Undifferentiated or anaplastic	0.1% (1)
T4	3.2% (40)	60-74	34.6% (426)	Missing	23.0% (283)	Unknown	4.1% (50)
Missing	3.9% (48)	>=75	27.4% (337)				

**Nodal status (N)**		**Metastasis status (M)**		**HER2-status (IHC)**			
N0	61.1% (753)	M0	76.5% (942)	Negative	80.8% (995)		
N1	19.7% (243)	M1	5.1% (63)	Positive	12.4% (153)		
N2	4.6% (57)	Missing	18.4% (227)	Missing	6.8% (84)		
N3	1.8% (22)						
Missing	12.7% (157)						

The overall three-year care costs stratified by disease stage are shown in Table 3. Total costs increased progressively from €23,907 in stage I to €86,247 in stage IV, representing a 3.6-fold increase. Hospitalization costs rose from €6,129 in stage I to €14,683 in stage IV (ratio = 2.4); also, outpatient costs increased from €10,413 in stage I to €23,372 in stage IV (ratio = 2.2). The most striking escalation was observed in in-hospital drug costs, which increased from €3,940 in stage I to €32,587 in stage IV (ratio = 8.3).

**Table 3:** Overall costs (euros) over three years from the diagnosis in BC patients (absolute values and row percentage). The ratio values refer to stage I.

	Hospitalization			Outpatients			In-hospital drugs			Outpatient drugs			Emergency room			Medical devices			Hospice			TOTALS	
Stage	Average cost	p	Ratio	Average cost	p	Ratio	Averagecost	p	Ratio	Average cost	p	Ratio	Average cost	p	-Ratio	Average cost	p	Ratio	Average cost	p	Ratio	Average cost	Ratio
Stage I	6129.44	25.6%	1.000	10413.06	43.6%	1.000	3939.58	16.5%	1.000	1707.96	7.1%	1.000	85.09	0.4%	1.000	1,629.28	6.8%	1.000	2.35	0.0%	1.000	23906.76	1.000
Stage II	8015.85	27.2%	1.308	12900.97	43.7%	1.239	5067.95	17.2%	1.286	2019.02	6.8%	1.182	124.71	0.4%	1.466	1,375.63	4.7%	0.844	10.39	0.0%	4.426	29514.52	1.235
Stage III	10330.69	22.9%	1.685	23698.37	52.4%	2.276	8311.47	18.4%	2.110	1946.55	4.3%	1.140	181.44	0.4%	2.132	714,82	1.6%	0.439	0.00	0.0%	0.000	45183.33	1.890
Stage IV	14683.43	17%	2.396	23372.49	27.1%	2.245	32587.19	37.8%	8.272	2127.52	2.5%	1.246	437.23	0.5%	5.138	11,339.28	13.1%	6.960	1,699.76	2.0%	724.204	86246.91	3.608
Missing	9164.30	23.9%	1.495	15385.83	40.1%	1.478	5600.00	14.6%	1.421	1725.04	4.5%	1.010	188.34	0.5%	2.213	6,126.71	15.9%	3.760	222.79	0.6%	94.923	38413.01	1.607
All Stages	7679.02	24.6%		13077.91	42%		5620.52	18%		1812.14	5.8%		131.33	0.4%		2,743.64	8.8%		100.25	0.3%		31164.82	

BC-specific costs stratified by disease stage over the three-year follow-up period are presented in Table 4. Total BC-specific costs increased from €14,613 in stage I to €65,808 in stage IV, corresponding to a 4.5-fold increase. The most substantial increase was documented for in-hospital drug expenses, which escalated from €1,499 in stage I to €31,331 in stage IV, representing a more than 20-fold increase (ratio = 20.9), driven by the extensive use of chemotherapy and targeted biological agents in advanced disease stages. Hospice care costs were almost entirely allocated to stage IV patients (€1,700; 2.6% of total BC-specific cost).

**Table 4:** Breast cancer-specific costs (euros) by item of costs over the three years after diagnosis (absolute values and row percentage). Emergency room, medical devices, and outpatient drug costs were not evaluated as specifically related to breast cancer.

	Hospitalization			Outpatients			In-Hospital drugs			Outpatient drugs			Emergency room			Medical device			Hospice			TOTALS	
Stage	Average cost	p	Ratio	Average cost	p	Ratio	Average cost	p	Ratio	Average cost	p	Ratio	Average cost	p	Ratio	Average cost	p	Ratio	Average cost	p	Ratio	Average cost	Ratio
Stage I	4713.34	32.3%	1.000	4978.29	34.1%	1.000	1498.64	10.3%	1.000	1707.96	11.7%	1.000	85.09	0.6%	1.000	1,629.28	11.1%	1.000	0,00	0%	_	14612.60	1.000
Stage II	6111.90	30.5%	1.297	6156.61	30.8%	1.237	4225.47	21.1%	2.820	2019.02	10.1%	1.182	124.71	0.6%	1.466	1,375.63	6.9%	0.844	6.68	0%	_	20020.03	1.370
Stage III	7137.16	24.4%	1.514	11495.14	39.2%	2.309	7818.63	26.7%	5.217	1946.55	6.6%	1.140	181.44	0.6%	2.132	714.82	2.4%	0.439	0.00	0%	_	29293.74	2.005
Stage IV	7608.28	11.6%	1.614	11264.04	17.1%	2.263	31331.45	47.6%	20.907	2127.52	3.2%	1.246	437.23	0.7%	5.138	11,339.28	17.2%	6.960	1,699.76	2.6%	_	65807.57	4.503
Missing	6077.12	23.7%	1.289	6571.64	25.7%	1.320	4772.15	18.6%	3.184	1725.04	6.7%	1.010	188.34	0.7%	2.213	6,126.71	23.9%	3.760	132.87	0.5%	_	25593.86	1.751
All Stage:	5543.16	27.1%		6097.50	29.8%		4037.06	19.7%		1812.14	8.9%		131.33	0.6%		2,743.64	13.4%		79.58	0.4%		20444.42	

The overall and BC-specific costs, stratified by HER2 status and disease stage, are reported in Table 5. Across all stages, HER2-positive patients incurred substantially higher costs compared to HER2-negative patients.

**Table 5:** Overall and BC-specific costs by HER2-status and stage (in euros). The ratio values refer to HER2 = NEG.

HER-2 Status	Stage I (*n* = 540)		Stage II (*n* = 290)		Stage III (*n* = 56)		Stage IV (*n* = 63)		Missing (*n* = 283)		All stages (*n* = 1232)	
	Cost	Ratio	Cost	Ratio	Cost	Ratio	Cost	Ratio	Cost	Ratio	Cost	Ratio
	**Overall costs**											
HER2 = NEG (*n* = 995)	22087.06	1.000	26469.11	1.000	42541.02	1.000	79468.24	1.000	32900.33	1.000	27812.79	1.000
HER2 = POS (*n* = 153)	38085.70	1.724	57778.26	2.183	59508.61	1.399	117595.37	1.480	60070.60	1.826	51735.42	1.860
Missing (*n* = 84)	16754.38	0.759	26142.01	0.988	17671.24	0.415	90856.44	1.143	45650.51	1.388	33511.55	1.205
All (*n* = **1232**)	23906.76	1.082	29514.52	1.115	45183.33	1.062	86246.91	1.085	38413.01	1.168	31164.82	1.121
	**BC-specific costs**											
HER2 = NEG (*n* = 995)	13219.86	1.00	17473.17	1.00	27943.86	1.00	59098.01	1.00	22068.30	1.00	17954.24	1.00
HER2 = POS (*n* = 153)	24837.59	1.879	41960.90	2.401	36763.00	1.316	98375.30	1.665	42181.39	1.911	35964.09	2.003
Missing (*n* = 84)	11496.26	0.870	21395.38	1.224	13729.84	0.491	66287.74	1.122	25770.00	1.168	21550.05	1.200
All (*n* = **1232**)	14612.60	1.105	20020.03	1.146	29293.74	1.048	65807.57	1.114	25593.86	1.160	20444.42	1.139

The mean overall cost was €27,813 in HER2-negative patients versus €51,735 in HER2-positive patients, corresponding to a 1.86-fold increase. Similarly, the mean BC-specific cost rose from €17,954 in HER2-negative to €35,964 in HER2-positive patients (ratio = 2.0). In stage IV, overall costs reached €79,468 for HER2-negative and €117,595 for HER2-positive patients (ratio = 1.5).

## Discussion

This population-based study offers empirical evidence concerning the comprehensive and breast cancer-specific treatment expenses observed over a three-year duration after the initial diagnosis of cancer, indicating that both disease stage and HER-status serve as determinants of healthcare expenditure.

### Cost of care by breast cancer stage

In real-world clinical practice, the present study further supports the key impact of BC stage at presentation on care expenditure. In 2018, a systematic review by Sun et al. examined 20 studies conducted in 10 countries with varying Gross domestic product and healthcare system management. In all included studies but one (Vietnamese) the BC care costs increased consistently from stage I to stage IV (7, 9). The mean treatment costs of stages II, III, and IV breast cancer were 32%, 95%, and 109% higher than those of stage I disease. However, at the single-country level, the cost difference between early (stage I-II) and advanced BC varied across studies and countries. Two relevant population-based studies by Mittmann et al. examined the stage-specific costs of breast cancer (3, 5, 6, 8). Both studies, in the same healthcare setting, confirmed that breast cancer costs increase depending on the stage at presentation. Notably, over the 10-year interval between the two studies, the gap in expenditure between low and high stages has consistently widened (2014 ratio stage IV: I = 2.5 versus 2024 ratio stage IV: I = 8.8). These findings probably stem from shifts in healthcare management and greater access to personalized therapies. Our data showed a stage IV:I ratio of roughly 4.5 for specific costs.

A systematic review by Franklin and colleagues involved 53 studies from North America, Australia, and Western Europe (2, 4), highlighting the substantial heterogeneity in cost estimates within and across countries, regarding cost perspective, methods of expenditure annotation, time horizon, and the reimbursement methods of different health care systems, making comparisons challenging, if not impossible. However, the conclusion was that the BC stage is the most significant factor influencing healthcare costs (2, 4). This issue is critically important because of its implications for the clinical and economic benefits of implementing both primary and secondary prevention strategies for breast cancer. By identifying breast cancer at earlier stages, healthcare systems can potentially achieve not only better survival outcomes but also significant cost savings (16, 21).

As expected, over the three years examined, the total expenditure per patient significantly exceeded the BC-specific costs (ratio = 1.55), even if the per-item cost values ranked in the same order for both overall and BC specific treatments. Outpatient costs, hospitalization, and in-hospital drugs were the most relevant, followed by expenditures for medical devices, outpatient drugs, emergency room, and hospice care. Interestingly, however, the ratio for stage IV to stage I differed according to the different cost distributions by stage. A UK study by Sun et al. found that surgery and radiotherapy accounted for the main costs of early-stage cancer management. Within the first year after diagnosis, these therapeutic procedures covered most of the hospital expenditure. Most early-stage procedures were delivered as day-case admissions, minimizing the care costs associated with inpatient care. This data confirms our findings, showing that hospitalization makes up one-third of total BC-specific healthcare costs in Stage I patients, while it accounts for only one-tenth of these costs in Stage IV patients. In contrast, according to the literature (2, 4, 5, 7, 9, 11), our study found that chemo- and targeted therapies are the main cost drivers in advanced stages.

### Cost care by HER2 status

This study demonstrated that cancers with HER2 receptor overexpression (HER2-positive) incur higher treatment costs than HER2-negative tumors. The current results consistently show that treatment costs for HER2-negative cancers are nearly half those for HER2-positive cancers. This economic trend was consistent across all cancer stages, emphasizing the substantial financial impact of tumor biology and personalized therapies from both clinical and economic viewpoints (17, 22).

Brezden-Masley et al. examined the economic burden associated with HER2-positive status (18, 19, 23, 24). These findings are consistent with those of Mahtani et al., who specifically evaluated the economic impact of HER2-targeted BC therapies (20, 25). Over three years, HER2 treatments contributed to a notable increase in costs. Addressing these findings critically, a systematic review by Franklin and colleagues noted that Mahatani’s estimates significantly exceeded the median costs reported in studies from other countries, where HER2-targeted therapies are categorized differently (2, 4). It is also essential to consider variations in therapeutic protocols across countries, including targeted treatments, when analyzing cost differences among studies (7, 9, 21, 26).

The cost analysis of BC care based on a regional Italian population might limit the study’s epidemiological representativeness and the generalizability of the results to settings with different healthcare systems, organizational structures, or resource availability. Future studies should aim to contextualize real-world cost data within different international frameworks, enabling more meaningful cross-country benchmarking and supporting the design of equitable, evidence-based cancer care policies at a global level.

Additionally, the study does not account for indirect costs, such as productivity losses and out-of-pocket expenses, which may lead to an underestimation of the societal burden of economic costs. These weaknesses may be mitigated by the low variability in patient care management (due to the consistently adopted shared diagnostic and therapeutic pathway at the regional level) and the reliable identification and calculation of expenditures per item. The study’s strengths also lie in the use of high-quality, population-level data, the efficient linkage across multiple administrative databases, and the detailed breakdown of costs by stage and HER2 status.

In conclusion, consistent with worldwide findings, this population-based study found that the cost structure of BC care increases with disease stage, with early-stage treatment primarily involving surgical and outpatient procedures. In contrast, advanced disease requires more expenditure on systemic therapies and hospital-based care. This evidence prioritizes the secondary prevention strategy not only for ethical and clinical reasons, but also due to its impact on saving care-related costs. Promoting early detection through effective screening programs can improve patient outcomes and optimize healthcare spending. This study also underscores the economic implications of tumor biology and therapeutic innovation, highlighting the financial impact of precision oncology in real-world clinical practice.

This evidence is key in supporting policymakers and healthcare providers in designing more efficient, equitable, and value-oriented cancer care and prevention strategies for breast cancer.

## Data Availability

The data analyzed in this study is subject to the following licenses/restrictions: The data supporting this study’s findings are held by the Veneto Epidemiological Registry and were used under license for this work. Further information is available from the corresponding author upon reasonable request. Requests to access these datasets should be directed to Manuel Zorzi manuel.zorzi@azero.veneto.it
